# Indigenous health and environmental risk factors: an Australian problem with global analogues?

**DOI:** 10.3402/gha.v7.23766

**Published:** 2014-04-29

**Authors:** Luke D. Knibbs, Peter D. Sly

**Affiliations:** 1 School of Population Health, The University of Queensland, Brisbane, Australia; 2 Children’s Health and Environment Program, The University of Queensland, Brisbane, Australia

**Keywords:** environmental health, Indigenous peoples, Australia, health, environment

## Abstract

Indigenous people experience poorer health than non-Indigenous people, and this well-described inequality has been observed in many countries. The contribution of different risk factors to the health ‘gap’ has understandably focussed on those factors for which there are sufficient data. However, this has precluded environmental risk factors – those present in air, water, food, and soil – due to a lack of data describing exposures and outcomes. These risk factors are demonstrably important at the global scale, as highlighted by the 2010 Global Burden of Disease study. Here, we describe how a greater focus on environmental risk factors is required in order to define their role in the Indigenous health gap. We use the Australian context as a case study of an issue we feel has global analogues and relevance. Suggestions for how and why this situation should be remedied are presented and discussed.

Access to clean air, soil, water, and food in a sustainable way is a fundamental tenet of human health. Without provision of these basic amenities, disease and inequality can manifest and perpetuate unchecked. This is hardly a novel proposition – Hippocrates and a procession of others since then have reminded us that when these critical foundations of health are compromised, the consequences for society can be profound. Yet in 2013, the world confronted the stark reality of a sizeable proportion of its population having no or infrequent access to clean water and sanitation, breathing air with potentially dangerous levels of vehicle and industrial emissions, and eating food contaminated by chemicals. Lead poisoning – recognised since Hippocrates’ time – continues to exact its toll on vulnerable children living in Africa (1). The visibility of environmental degradation and its consequences for human health have never been greater.

Many of the Millennium Development Goals (MDGs) target problems with environmental causes. The ecological nexus between humans and the physical environment is one of the pillars on which our health and wellbeing rests. It is therefore unsurprising that indoor and outdoor air pollution feature in the top 10 risk factors in the global burden of disease, while lead, poor sanitation, and water quality are all present in the top 30 (2). However, some populations are more susceptible to these risk factors than others (3).

Indigenous peoples worldwide bear a disproportionate share of the burden of disease. The reasons for this are complex, but marginalisation following colonisation of their traditional homelands are recurring themes that have initiated a legacy of inequity and disadvantage that is unfortunately pronounced among the near 400 million Indigenous people worldwide today (4).

Indigenous Australians (Aboriginal and Torres Strait Islander peoples) experience a markedly greater disease burden compared with non-Indigenous Australians, and newborn Indigenous Australians are expected to live a decade less than their non-Indigenous counterparts (5). The health ‘gap’ is largely attributable to non-communicable diseases (NCDs), about half of which can be explained by established risk factors including tobacco and alcohol use, obesity, physical inactivity, and inadequate fruit and vegetable intake (6). Efforts to quantify the extent to which other factors – such as those in the environment – contribute to the burden of disease and health gap have been hampered by a lack of data describing exposures and outcomes among the Indigenous population (6).

‘Environmental’ risk factors are often an ambiguous concept, as the malleable definition of environment can take on vastly different meanings depending on the context. The more traditional and narrower scientific definition encompasses biological, chemical, and physical agents encountered in the natural and built environment that are capable of causing harm. Conversely, environmental risk factors can be taken to represent anything a person encounters in his/her life that is not genetic, whether physical, social, behavioural, economic, cultural, or any combination of these (7). While the latter definition is valid, there is a lot we do not know about the traditional environmental risk factors on Indigenous people’s health, let alone their complex interactions with other risk factors.

Improved understanding of the environmental risk factors experienced by Indigenous Australians will better define their role in cardiovascular, endocrine and neurodevelopmental disease, and cancer, all of which can have environmental aetiology and are substantial contributors to the disease burden. The issue currently faced is how to understand the true nature of environmental risks to Indigenous Australians, which are demonstrably important at a worldwide scale. It is also a case study relevant to other Indigenous communities globally.

## The Australian context

### Ambient and indoor air pollution

Air pollution describes the complex mixture of gaseous and particulate contaminants in the atmosphere. There is a small body of evidence that shows that the effects of ambient PM_10_ (particles <10 µm) from bushfires on respiratory and cardiovascular illnesses are greater in Indigenous people compared with non-Indigenous people (8). Similarly, pregnant Indigenous women may be at greater risk of pre-eclampsia due to ambient traffic-related air pollution than non-Indigenous women (9). Exposure to second-hand smoke indoors places Indigenous children at higher risk of developing otitis media (10).

### Asbestos

Asbestos is a naturally occurring fibrous mineral that was found in many domestic, commercial, and industrial applications throughout the 20th century. Inhalation of asbestos fibres is the overwhelming risk factor for malignant mesothelioma, an aggressive and fatal cancer, as well as lung cancer and asbestosis. Australia has one of the world’s highest incidences of malignant mesothelioma. Indigenous people in historic asbestos mining regions had the world’s second-highest crude incidence rate of malignant mesothelioma (250 per million person-years) in the 1990s – about 10 times the national rate (11, 12).

### Weather and climate

Weather, especially temperature and rainfall, can exert many direct and indirect effects on health. Heat waves and cool spells can cause fatal hyper- and hypothermia, respectively, while severe weather and flooding can pose an immediate threat through injury or drowning. More expansive are their many indirect effects on health via vector- and water-borne infections, crop yields, and population displacement (13).

In the tropical Kimberley area of northern Western Australia, a marked increase in the proportion of very low birth weight (<1,500 g) Indigenous babies was observed in the ‘wet’ season (January to June) compared to the ‘dry’ season (14). Many infectious diseases exhibit a strong seasonality, especially in tropical locations, but the specific effects on Indigenous Australians are not well-documented. The role of weather and climate on Indigenous health is poorly defined, and this lack of information becomes starker when the prevailing backdrop of climate change is considered.

### Contaminated water and land

Remote Indigenous communities can be particularly susceptible to water contamination. Stagnant water can promote mosquito breeding and facilitate transmission of vector-borne diseases. Insufficient access to clean water and sewerage systems remain contributing factors to skin, eye, and diarrhoeal illnesses among Indigenous communities, especially children (15, 16).

Mining conducted near Indigenous land can leave a legacy of copper sulphide contamination causing substantial impacts on ecosystems with cultural and environmental significance. Australia’s vast deposits of uranium have been mined in rural areas located on or near traditional Indigenous lands. Some traditional foods (freshwater mussels, turtles, and fish) are strong bio-accumulators of ionising radiation from mining waste (17). Nuclear weapons tested during the post–World War II period in the vicinity of Indigenous communities resulted in the presence of residual radionuclides for several decades. However, the effects of radioactivity on the health of Indigenous peoples are unknown.

Indigenous children in mining communities have been shown to be at greater risk of abnormally high levels of lead in blood compared to their non-Indigenous counterparts, which was attributed to poorly maintained housing and bare soil (18). Iron deficiency due to poor diet may also promote lead uptake.

Cadmium has detrimental renal effects, and it is present in several traditional Indigenous sea foods such as turtle, dugong, and clams. The high prevalence of diabetes among Indigenous people coupled with dietary exposure to cadmium can exacerbate diabetic nephropathy (19).

## What we do and do not know

There is scarce information on the association between environmental risk factors and Indigenous peoples’ health in Australia. Most of the very limited work has quantified environmental exposures without linking these to health outcomes. This is concerning given the role of environmental risk factors in many of the communicable, and especially non-communicable, diseases that contribute to the Indigenous disease burden (4). There is also a pronounced lack of information on gradients of exposure and health effects across urban, rural, and remote areas, which is a crucial distinction as most Indigenous people do not live in cities.

We know that environmental risk factors are important globally, and we know only half of the Indigenous health gap can be attributed to non-environmental risks. We suspect that Indigenous people may be more susceptible to the health effects of some environmental risk factors. But we do not know how much disease they cause. It is important not to over- or understate the effects these risk factors may have, because there is simply too little information for unequivocal conclusions to be drawn.

## What we can do about it

We need to better understand how an Indigenous Australian’s health can potentiate the extent to which they are susceptible to environmental risk factors, and what role social determinants of health play in establishing this relationship. Properly delineating the role of environmental risk factors will enable their inclusion and relative place in the spectrum of contributors to Indigenous disease to be determined. A deeper understanding is the first step towards prioritising research, policy, and interventions. The well-described tools at our disposal, such as health impact assessment and comparative risk assessment, have much to offer in achieving this (20). Understanding the source and control of all relevant environmental risk factors will mean they can be more effectively targeted and prioritised.

## Global relevance

Australia is a highly developed country that performs admirably on most measures of human and economic development. Yet, its record on Indigenous health leaves much to be desired. This is reflected in our lacklustre understanding of environmental contributors to the Indigenous disease burden. If a country such as Australia that is rich in human and natural resources struggles to make inroads on this issue, it does not bode well for more poorly resourced settings where the burden is likely to be the greatest (3). The Australian Indigenous people make up less than 0.2% of the world’s Indigenous population, but the issues faced in defining the role of environmental risk factors are symptomatic of a wider global problem. Adverse health effects due to environmental risk factors have been described in numerous Indigenous populations around the world (21–25). Notwithstanding methodological differences and limitations, there is a modest but accumulating body of evidence about these risks and how they compare with those in non-Indigenous people. This provides a good foundation on which to build the more extensive studies required to address the issue.

The diverse nature of environmental risk factors requires an equally diverse interdisciplinary approach to their quantification and control. Researchers, health care professionals, non-government organisations, and policy-makers are all in a position to contribute towards redressing the situation. If we are to understand the effect of the environment on the health of Indigenous people, we first need to make a clarion call to the people who have the greatest capacity to undertake this important work.
